# Luteolin impacts deoxyribonucleic acid repair by modulating the mitogen-activated protein kinase pathway in colorectal cancer

**DOI:** 10.1080/21655979.2022.2066926

**Published:** 2022-04-27

**Authors:** Yelin Song, Jie Yu, LingLing Li, Lei Wang, Liangle Dong, Guangmin Xi, Yun Jing Lu, Zuowei Li

**Affiliations:** a Department of cardiovascular medicine, Qingdao Hospital of Traditional Chinese Medicine, Qingdao, Shandong, China; b Cardiovascular disease department, Shandong University of Traditional Chinese Medicine, Jinan, Shandong, Chinas; c Digestive System Department, Chengyang District People’s Hospital, Qingdao, Shandong, China; d Department of Respiratory Medicine, Jinling Hospital, Nanjing University School of Medicine, Nanjing, China; e College of Life Science, Qi Lu Normal University, Jinan, Shandong, China; f Medical Department, People’s Hospital of Chengyang, Qingdao, Shandong, China

**Keywords:** Luteolin, colorectal cancer, DNA damage, MAPK pathway, cisplatin, selumetinib

## Abstract

This study aimed to investigate the effects of luteolin on colorectal cancer (CRC) and explore its underlying mechanism. HCT-116 and HT-29 cells were treated with luteolin, cisplatin, or selumetinib. The cell survival, cell proliferation, apoptosis and cell cycle distribution, and DNA damage were detected using Cell Counting Kit-8, colony formation, flow cytometry, and immunofluorescence staining analysis, respectively. Western blotting was used to detect the expression of apoptosis-related, cycle-related, DNA-damage-related, and mitogen-activated protein kinase (MAPK) pathway-related proteins. Luteolin showed inhibitory effects on cellular growth by reducing cell survival and proliferation, inducing apoptosis and DNA damage, and arresting the cell cycle in a concentration-dependent manner in HCT-116 and HT-29 cells. Meanwhile, luteolin increased the expression of pro-apoptotic proteins, p-CHK1 (central to the induction of cell cycle arrest), and DNA excision repair protein and decreased anti-apoptotic proteins, G2-M phase-related proteins, and DNA repair proteins. The combination of cisplatin and luteolin significantly decreased cell survival and increased the apoptosis rate of HCT-116 and HT-29 cells compared with cisplatin alone. Bioinformatic analysis using the Comparative Toxicogenomics Database and STITCH and MalaCards databases showed that the MAPK pathway is involved in the pharmacology of luteolin. Furthermore, western blotting demonstrated that luteolin plays an inhibitory role by suppressing the MAPK signaling pathway in CRC, which is enhanced when combined with selumetinib. Luteolin can also prevent tumourigenesis in CRC in vivo. In conclusion, luteolin suppressed cell proliferation, blocked the cell cycle, and induced DNA damage and apoptosis progression in CRC cells by mediating the MAPK pathway

## GRAPHICAL ABSTRACT

**Figure:**
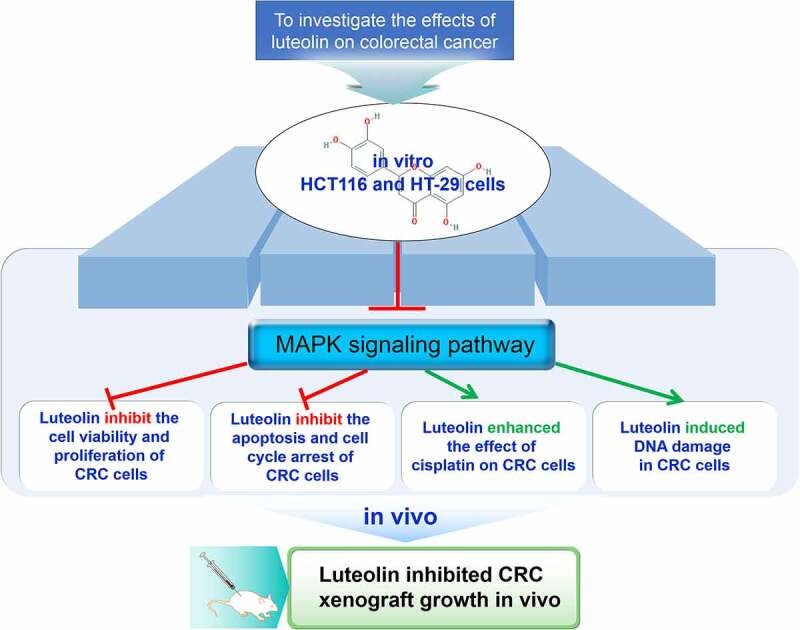

## Highlights

- Luteolin inhibits the viability and proliferation of CRC cells.
- Luteolin induces the apoptosis and cell cycle arrest of CRC cells.
- Luteolin enhances the effect of cisplatin on CRC cells
- Luteolin induces DNA damage in CRC cells.
- Luteolin inhibits the MAPK signalling pathway in CRC cells.
- Luteolin inhibits CRC xenograft growth in vivo.

## Introduction

Cancer remains one of the deadliest diseases in the world [1]. Colorectal cancer (CRC) is the third most common malignant tumor and the second leading cause of cancer-related death globally [2]. The incidence rate of CRC is rapidly increasing in both developed and developing countries owing to economic development and changes in diet and lifestyle, especially the new trend in young adults. There are 1.8 million new CRC cases worldwide every year, which is predicted to increase to >2.2 million by 2030 [3]. Only approximately 40% of CRC cases are genetic and have a family history of CRC, while most of the other 60% cases are sporadic, and genetic mutations increase the risk of CRC [4]. At present, several therapeutic methods for CRC, such as surgery, radiotherapy, adjuvant chemotherapy, receptor-targeted therapy, and various combinations of monoclonal antibodies, have achieved significant progress in the clinical setting [2,5]. However, CRC remains one of the most life-threatening malignant tumors, especially in patients with advanced CRC, with a 5-year survival rate of only 12% [6]. Therefore, it is necessary to identify new alternatives to CRC-preventive drugs.

In recent years, plant derivatives have become important sources for the development of new drugs. Luteolin (3,4,5,7-tetrahydroxyflavone) is a flavonoid that is abundant in vegetables (e.g. broccoli, carrot, celery, and pepper), fruits (e.g. blueberry, blueberry, and blackberry), and herbal medicines (e.g. honeysuckle, *Schizonepeta tenuifolia*, and *Prunella vulgaris*) [7]. Luteolin has a wide range of biological activities and functions, including immunoregulation, liver protection, anti-inflammatory, antioxidant, anti-allergy, antiviral, anti-atherosclerosis, anti-diabetes, and anti-tumor [8–10]. Among them, the anti-tumor effect has attracted extensive attention because it is an inhibitor of topoisomerases I and II [11,12], which makes it a leading anticancer compound [13,14].

Luteolin has been widely studied in several cancers, including melanoma [15], lung adenocarcinoma [16], gastric cancer [17], esophageal cancer [18], placental choriocarcinoma [19], breast cancer [20], and hepatocellular carcinoma [21]. Luteolin has also been studied in CRC, interfering with the cell cycle; inducing apoptosis; and inhibiting cell proliferation, angiogenesis, epithelial-to-mesenchymal transition, and metastasis [22,23]. A number of studies have shown that exposure of cancer cells to chemotherapeutic drugs can result in DNA damage. However, the detailed mechanism underlying the action of luteolin on DNA damage in CRC cells remains unclear. In this study, we investigated the anti-proliferative and apoptotic activities of luteolin in the human CRC cell lines HCT-116 and HT-29. In addition, the effects of luteolin on the cell cycle, DNA damage, and mitogen-activated protein kinase (MAPK) signaling pathway were investigated, highlighting the underlying molecular mechanism involved in the behavior of CRC cells.

This study aimed to investigate the effects of luteolin on CRC and explore its underlying mechanism. We evaluated luteolin as a potential therapeutic agent for CRC. Our results suggest that luteolin could suppress the cell viability and proliferation, block the cell cycle, and induce DNA damage and apoptosis progression in CRC cells by mediating the MAPK pathway.

## Materials and methods

### Cell cultures and treatment

Human CRC cell lines (HCT-116 and HT-29) were obtained from the American Type Culture Collection (Rockville, MD, USA). Cells were routinely cultivated in Dulbecco’s Modified Eagle’s Medium (DMEM; HyClone, USA) containing 10% fetal bovine serum (FBS; Gibco, USA) in 10-cm plates in a humidified atmosphere of 95% air and 5% CO_2,_ at 37°C. While achieving approximately 90% confluency, the cells were routinely cultured for 6th–10th passages using 0.25% trypsin-EDTA for the following treatment [24].

After all-night cultivation allowing for adherence, the cells were exposed to different concentrations of luteolin (Yuan ye Biological Technology, Shanghai, China; diluted in 500 mM of dimethyl sulfoxide [DMSO]; groups treated with 0, 10, 20, and 40 μM of luteolin) and/or 5 μM of cisplatin (QILU Pharmaceutical, Shandong, China; CIS group, CIS+10 μM, CIS+20 μM, and CIS+40 μM groups) and/or 100 nM of selumetinib (MEK inhibitor AZD6244; selumetinib and selumetinib+40 μM groups) or 0.2% DMSO (Hebei Bio-high Technology Deve Co., LID, Hebei, China; control group) in DMEM containing 5% FBS.

### Cell viability assay

Cell Counting Kit-8 (CCK-8) assay was performed to identify the effects of luteolin and/or cisplatin on cell viability. Briefly, the day before the experiment, HCT-116 and HT-29 cells were inoculated into a 96-well plate (5 × 10^3^ cells/well) and treated with various concentrations of luteolin (0, 10, 20, and 40 μM) in the presence or absence of 5 μM of cisplatin for 24 h. Subsequently, after removing the medium, 10 μL of CCK-8 reagent (GenePharma, Shanghai, China) and 100 µL of phosphate-buffered saline (PBS) were added to each well and cultured for an additional 3 h at 37°C [25]. A plate reader (Molecular Devices, CA, USA) was used to measure absorbance at 450 nm (OD_450_) at 24, 48, and 72 h. The design formulas for cell survival (%) are presented as (OD_control_-OD_drug_)/OD_control_ × 100% [26].

### Colony formation assay

Cell proliferation was detected using a colony formation assay. Briefly, HCT-116 and HT-29 cells were seeded in a 6-well plate (1 × 10^2^ cells/well) coated with Matrigel. The cells were then treated with various concentrations of luteolin (0, 10, 20, and 40 μM) in complete medium for 2 weeks, which was replaced every 3 days. Subsequently, colonies with greater than 50 cells were fixed with 4% formaldehyde for 15 min at 37°C and stained with crystal violet (Merck, Germany) for 10–20 min. Finally, the cell colonies were captured by cameras and counted from five random fields using ImageJ software (Bethesda, MD, USA) [25,27].

### Apoptosis analysis

The Annexin V-PI kit (Beyotime Institute, Nantong, China) was used for staining, and flow cytometry was used to detect cell apoptosis. Briefly, HCT-116 and HT-29 cells were incubated with luteolin (0, 10, 20, and 40 μM) in the presence or absence of 5 μM of cisplatin for 48 h. Subsequently, cells were cultivated with 10 μL of Annexin V in the dark for 15 min, followed by the addition of 10 µL of propidium iodide (PI) to the cell suspension. Finally, the apoptosis rate of the cells was detected using a FACScan flow cytometer (BD Biosciences, CA, USA) [28,29].

### Cell cycle assay

HCT-116 and HT-29 cells (3 × 10^5^ cells) were seeded in 6-well plates and cultivated in media with different concentrations of luteolin (0, 10, 20, and 40 µM) for 48 h. The cells were collected, washed three times with PBS, and fixed with 0.7 mL of 75% pre-cooled ethanol at −20°C overnight. Subsequently, after rinsing with PBS, the cells were re-suspended in 500 μL of buffer containing 10 μL of RNAse (Sigma) and 25 µL of PI (BD Biosciences) and incubated at room temperature for 30 min in the dark. The percentage of cells in different phases (G1, S, and G2) of the cell cycle was detected by flow cytometry (BD Biosciences, Franklin, NJ, USA) [28,30].

### Immunofluorescence staining analysis

HCT-116 and HT-29 cells (1 × 10^6^ cells/well) were inoculated into 6-well plates with a cover glass for 24 h. Then, the cells were treated with different doses of luteolin (0, 10, 20, and 40 µM). After 48 h of treatment, the cells were harvested and fixed in 4% paraformaldehyde at 4°C for 15 min at atmospheric temperature, washed three times with PBS, and permeabilised with 0.2% Triton X for 2 h. Subsequently, the cells were incubated with primary antibodies against γH2aX (1:1000; Upstate Biotechnology, Temecula, CA, USA) for 2 h, washed three times with PBS, and incubated with secondary Alexa Fluor 488-conjugated anti-mouse antibody (1:1000; Invitrogen, Carlsbad, CA, USA) for 1 h. After washing, the cells were overlaid with 10 μL/mL of DAPI (Sigma-Aldrich) for 20 min. Finally, the cells were sealed with 90% glycerol, and γH2aX foci were observed using a fluorescence microscope (Nikon, Japan) [31,32].

### Western blot analysis

HCT-116/HT-29 cells (2 × 10^6^ cells) were seeded in a 100-mm dish. After treatment with luteolin and/or selumetinib for 48 h, cells were collected in PBS and lysed with radioimmunoprecipitation assay (RIPA) buffer (Beyotime Institute of Biotechnology, Beijing, China) on ice for 30 min, and lysates were harvested by centrifugal separation. Protein concentration was determined using the BCA Protein Assay Kit (Takara). Identical amounts of protein were separated using 10% sodium lauryl sulfate-polyacrylamide gel electrophoresis and then transferred to polyvinylidene difluoride membranes (Millipore, USA). The membranes were clogged with 5% nonfat dry milk for 1 h at room temperature. The primary antibodies BCL-2, BAX, cleaved caspase-3, cyclin B1, p-CHK1, CDC2, XRCC1, LIG1, HMGB1, ERCC3, p-MEK1, MEK1, p-ERK1/2, ERK1/2, and GAPDH (1:1000 dilution, Sigma-Aldrich) were added after overnight incubation at 4°C. After three washes with TBST, the membranes were incubated with anti-mouse horseradish peroxidase (HRP)-linked secondary antibodies (Beyotime Institute of Biotechnology, Beijing, China) for 1 h. The intensity of protein expression was measured using an enhanced chemiluminescence reagent (Thermo Fisher Scientific, USA) [31,32].

### Bioinformatic analysis

Using COADREAD, luteolin, and COADR as keywords, the signaling pathways were searched in databases, including the Comparative Toxicogenomics Database (CTD, http://ctdbase.org/) and STITCH (http://stitch.embl.de/) and MalaCards (https://www.malacards.org/) databases. The Kyoto Encyclopedia of Genes and Genomes (KEGG) analyses were conducted using the R package ‘ggplot2’.

### Xenograft mouse model

To establish a subcutaneous xenograft mouse model, a total of 12 5-week-old female BALB/c nude mice were obtained from Gem Pharmatech (Nanjing, China) and reared under 25–27°C, 45%–50% humidity, and standard 12-h light/dark cycle conditions with food and water accessibility. HCT-116 cells were collected at the logarithmic growth phase and suspended in a single-cell suspension (3 × 10^7^/mL) in serum-free RPMI-1640 medium. Then, 0.2 mL of the prepared cell suspension was subcutaneously inoculated into the dorsal flanks of the nude mice. When tumors reached suitable volumes, all animals were randomly assigned to three groups with four mice in each group. Tail vein injections were performed for 13 consecutive days with the following schedule: control group, PBS; high-dose group, luteolin at 40 mg/kg/d; and low-dose group, luteolin at 20 mg/kg/d. The diameter of the long/short axis and daily animal activities were recorded. Individual tumors were weighed after all mice were sacrificed, and xenograft tumor tissues were obtained and photographed. Furthermore, the volumes were calculated using the following formula: V (mm3) = length (L) × width (W)2/2. All procedures were conducted aseptically in accordance with the Guide for the Care and Use of Laboratory Animals of our hospital.

### Immunohistochemistry analysis

Ki67 expression in the prepared tumor tissues was assessed by immunohistochemistry. Briefly, the prepared tissues were fixed in formalin, embedded in paraffin, and sliced into 3-μm-thick sections. The sections were heated at 60°C for 2 h, dewaxed in xylene, and rehydrated in graded ethanol. Subsequently, citrate buffer was heated for 30 min to induce antigen retrieval. The sections were treated with 3% H2O2 for 15 min to block endogenous peroxidase activity. The sections were incubated with primary antibodies (Ki67, #MA5-14,520) at 4°C overnight. Thereafter, HRP-labeled secondary antibody (#A16096) was incubated at 25°C for 1 h. Finally, the sections were stained with diaminobenzidine (#P0202, Beyotime), and the nuclei were counterstained with hematoxylin (#C0107, Beyotime). For the negative control (NC) group, antibodies were replaced with PBS. The results were visualized and photographed under a microscope (Olympus, Tokyo, Japan).

### TUNEL staining

Apoptosis in tissue sections was detected using TUNEL assays. According to the manufacturer’s specifications, a one-step TUNEL apoptosis assay kit (Beyotime, Jiangsu, China) was used to treat paraffin sections. Images were captured using a photomicroscope (Carl Zeiss, Oberkochen, Germany). TUNEL-positive cells were quantified and counted in more than three random fields.

### Statistical analysis

All data were analyzed using SPSS (version 20.0; Chicago, IL, USA). All experiments were conducted in triplicate, and all data are expressed as mean ± standard deviation. Differences between two groups were determined using Student’s t-test. Statistical significance was set at p < 0.05.

## Results

In this study, we investigated the effects of luteolin on CRC and explored the underlying mechanism. Our results show that luteolin can suppress cell proliferation, block the cell cycle, and induce DNA damage and apoptosis progression in CRC cells by mediating the MAPK pathway.

### Effects of luteolin on CRC cells

**Figure 1.**
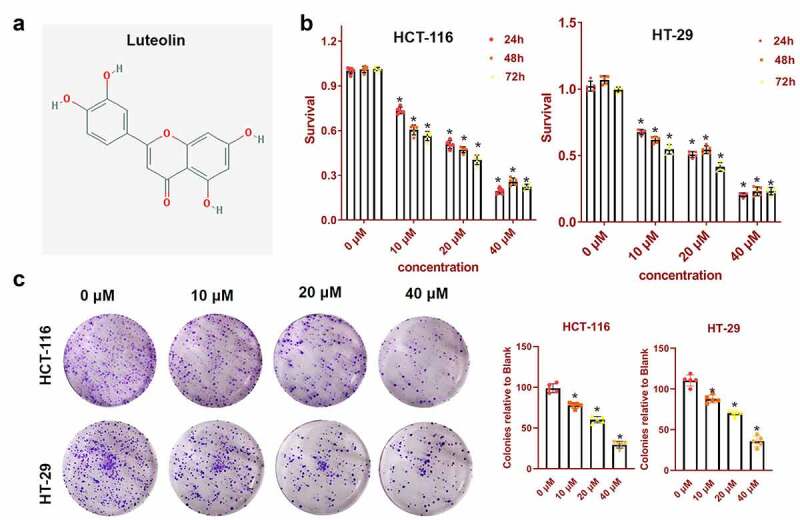
Luteolin inhibits the cell viability and proliferation of colorectal cancer cells. (a) The structure of luteolin. (b) The viability of HCT-116 and HT-29 cells is assessed by cell counting Kit-8 assay after treated with different concentrations of luteolin (0, 10, 20, and 40 μM) for 24, 48, and 72 h. (c) The proliferation of HCT-116 and HT-29 cells treated with different concentrations of luteolin (0, 10, 20, and 40 μM) is confirmed using a the soft agar assay. histogram shows the colonies relative to blank. *P < 0.05, compared with the 0-μM luteolin group.

The structure of luteolin is shown in Figure 1a. To investigate the cytotoxicity of luteolin, the CCK-8 assay was carried out to determine the viability of HCT-116 and HT-29 cells after treatment with a gradual increase in the dose of luteolin for 24, 48, and 72 h. Luteolin gradually decreased cell viability in a dose-dependent manner (0, 10, 20, and 40 μM), and the 24- and 48-h treatments with luteolin were less toxic than the 72-h treatment, especially in HCT-116 cells (Figure 1b) with time-concentration dependence. Non-anchored growth is a potential indicator of tumourigenesis and metastasis. The effect of luteolin on colony formation by HCT-116 and HT-29 cells was confirmed using a soft agar assay. As shown in Figure 1c, luteolin dramatically reduced the colony formation viability in a dose-dependent manner. Collectively, luteolin suppressed the viability, proliferation, and tumourigenicity of CRC cells.

### Luteolin induced the apoptosis and cell cycle arrest of CRC cells

**Figure 2.**
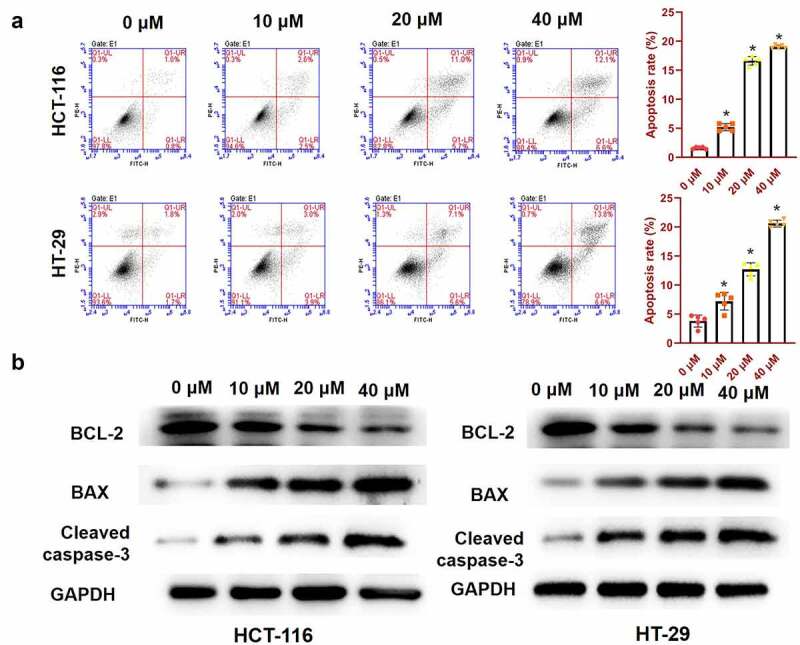
Luteolin induces the apoptosis and cell cycle arrest of colorectal cancer cells. (a) Flow cytometry identifies apoptotic cells following treatment with 0, 10, 20, and 40 μM of luteolin, and then the cell apoptosis rate is assessed. (b) Western blotting assays are used to detect the mitochondrial apoptosis-related proteins. (c) Cell cycle analysis is performed in HCT-116 and HT-29 cells treated with 0, 10, 20, and 40 μM of luteolin, and the percentage of cells in the G1, S, and G2 phases is assessed. (d) The G2-M phase-related protein expression is detected by western blotting. *P < 0.05, compared with the 0-μM luteolin group.

As luteolin could inhibit cell proliferation, we investigated whether it could induce cell apoptosis. Apoptosis of HCT-116 and HT-29 cells after 48 h of treatment with luteolin was determined by flow cytometry. As shown in Figure 2a, the level of apoptosis was gradually elevated in cells treated with 10, 20, and 40 μM of luteolin compared with that in the 0-μM luteolin group. To thoroughly investigate the potential mechanisms involved in the effect of luteolin on cell apoptosis, the expression levels of anti-apoptotic proteins (BCL-2) and pro-apoptotic proteins (BAX and cleaved caspase-3) were measured by western blotting. The results indicated that luteolin led to a statistically significant increase in the protein expression of BAX and cleaved caspase-3, whereas it resulted in a dramatic decrease in BCL-2 expression in HCT-116 and HT-29 cells in a dose-dependent manner HCT-116HT-29 (Figure 2b). These results showed that luteolin suppressed the growth of CRC cells by inducing apoptosis.

To further examine whether the inhibitory effects of luteolin on cell growth were related to the abduction of cell cycle arrest, we used flow cytometry to investigate the cell cycle distribution in luteolin-treated cells. As shown in Figure 2c, luteolin induced a dramatic alteration in the cell cycle distribution in both HCT-116 and HT-29 cell lines treated with luteolin, as manifested by the percentage of cells in G2, which increased in a dose-dependent manner (0, 10, 20, and 40 μM). Correspondingly, western blotting showed that the levels of G2-M phase-related proteins, including cyclin B1 and CDC2, decreased, whereas those of p-CHK1 (central to the induction of cell cycle arrest) increased in a concentration-dependent manner (Figure 2d). These results showed that luteolin could induce cell cycle arrest at the G2 phase, resulting in the inhibition of CRC cell proliferation.

### Luteolin enhanced the effect of cisplatin on CRC cells

**Figure 3.**
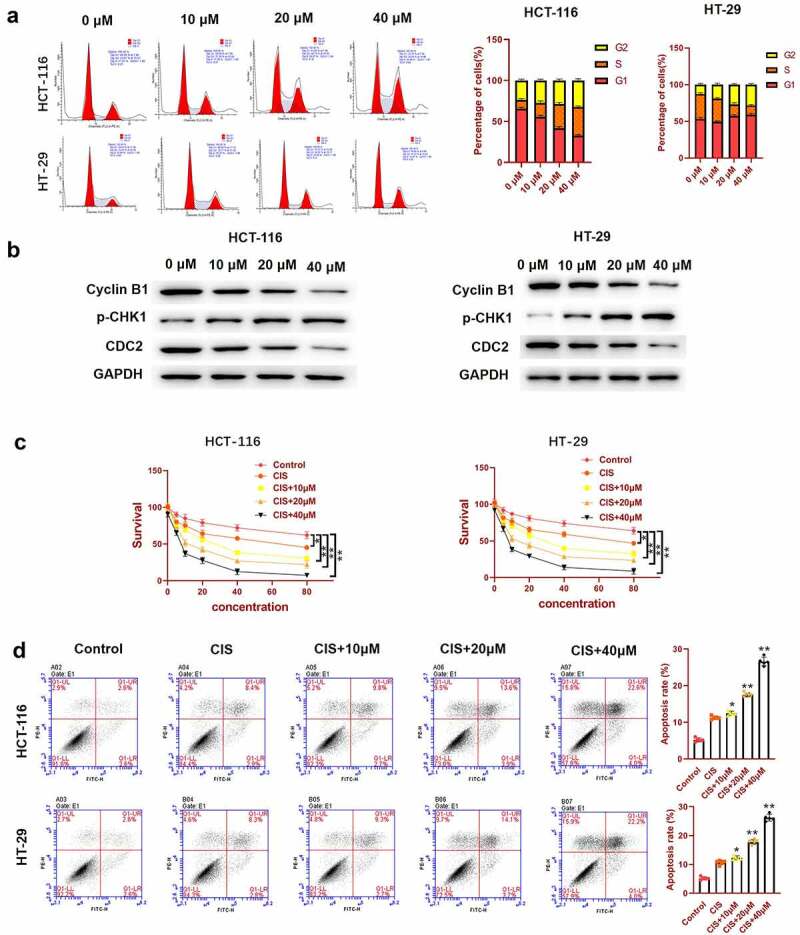
Effects of the therapeutic agent cisplatin with or without luteolin on survival and apoptosis of colorectal cancer cells. HCT-116 and HT-29 cells are treated with indicated concentrations of cisplatin with/without luteolin for 48 h. (a) Cell viability is evaluated by Cell Counting Kit-8 assay. (b) Cell apoptosis is evaluated by flow cytometry. CIS, cisplatin. *P < 0.05, **P < 0.01, compared with the CIS group.

To study the combined effect of luteolin and cisplatin on HCT-116 and HT-29 cells, cell viability and apoptosis were evaluated by CCK-8 assay and flow cytometry following a 48-h single treatment of cisplatin or combinatorial treatment with luteolin (10, 20, and 40 μM). As shown in Figure 3a, cisplatin alone remarkably decreased the survival rate of HCT-116 and HT-29 cells. Cells treated with the combination of cisplatin (5 μg/mL) and luteolin showed a more significant decrease in cell survival in a dose-dependent manner than cells treated with cisplatin alone. Meanwhile, the apoptosis rate of HCT-116 and HT-29 cells increased by cisplatin alone was further aggravated by co-treatment with luteolin in a dose-dependent manner (Figure 3b). These results indicate that luteolin has a synergistic or additive effect on the growth of CRC cells when combined with cisplatin.

### Luteolin induced DNA damage in CRC cells

**Figure 4.**
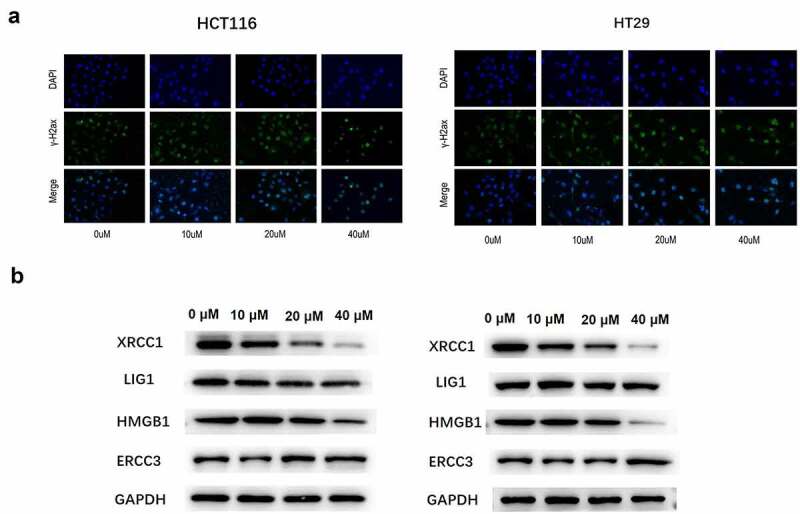
Luteolin induces DNA damage in colorectal cancer cells. (a) Immunofluorescence staining of HCT-116 and HT-29 cells using anti-γ-H2AX antibody and DAPI. Cells are treated with 0, 10, 20, and 40 µM of luteolin for 48 h. (b) DNA (excision) repair proteins are detected by western blotting.

Double-strand breaks are a type of DNA damage that can be determined by measuring the number of γH2aX foci [33]. In the present study, we first explored luteolin-induced DNA damage by measuring the number of γH2aX foci. HCT-116 and HT-29 cells were exposed to different doses of luteolin (0, 10, 20, and 40 µM) for 48 h, and the number of γH2aX foci was measured by immunofluorescence staining. As shown in Figure 4a, the number of γH2aX foci gradually increased in a dose-dependent manner after treatment with luteolin. Furthermore, the levels of DNA repair proteins (HMGB1, LIG, and XRCC1) and DNA excision repair protein (ERCC-3, a tumor-preventing gene) were detected by western blotting. The results showed that luteolin decreased the protein levels of HMGB1, LIG, and XRCC1 but increased the level of ERCC-3 in HCT-116 and HT-29 cells in a dose-dependent manner (Figure 4b).

### Luteolin attenuated MAPK signaling pathways in CRC cells

**Figure 5.**
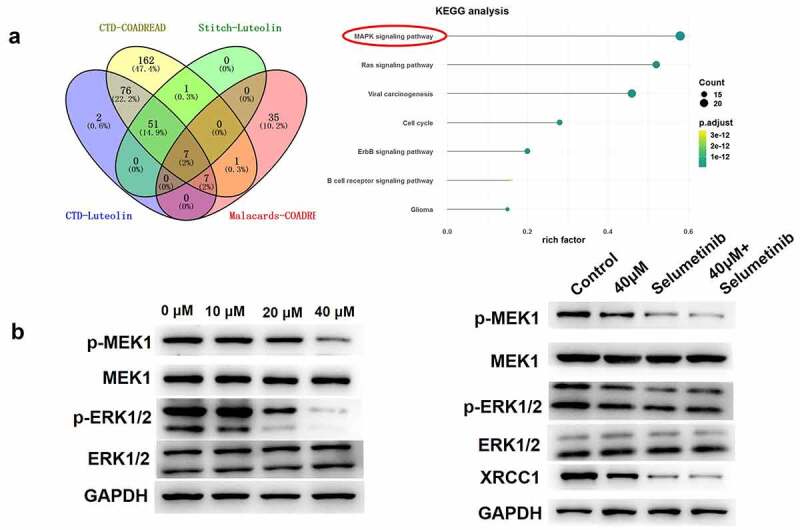
Luteolin inhibits the mitogen-activated protein kinase (MAPK) signaling pathway in colorectal cancer cells. (a) Bioinformatic analysis identifies the key pathways associated with the pharmacology of luteolin. (b) The expressions of different proteins (p-ERK1/2, ERK1/2, MEK1, and p-MEK1) related to the MAPK signaling pathway are detected by western blotting in luteolin-/selumetinib-treated HCT-116 cells.

To elucidate the mechanism by which luteolin influences the behavior of CRC cells, the corresponding targets of luteolin were identified based on network pharmacology using the CTD and STITCH and MalaCards databases. The KEGG pathway enrichment analysis indicated that seven key pathways were likely related to the pharmacology of luteolin (Figure 5a). Subsequently, the effect of luteolin on the MAPK signaling pathway was investigated, as it has been reported that this pathway is inhibited in CRC cell [34]. As shown in Figure 5b, western blot analysis indicated that the levels of MAPK pathway-related proteins, including MEK1 and ERK1/2, were not significantly altered, whereas the levels of p-MEK1 and p-ERK1/2 were decreased in a dose-dependent manner in luteolin-treated HCT-116 cells. Furthermore, the combination of selumetinib and luteolin (40 µM) significantly decreased the phosphorylation levels of MEK1 and ERK1/2 compared with selumetinib or luteolin alone and decreased the level of the DNA repair protein XRCC1 (Figure 5b). Taken together, luteolin plays a suppressive role in the behavior of CRC cells by inhibiting the MAPK signaling pathway.

### Luteolin inhibited CRC xenograft growth in vivo

**Figure 6.**
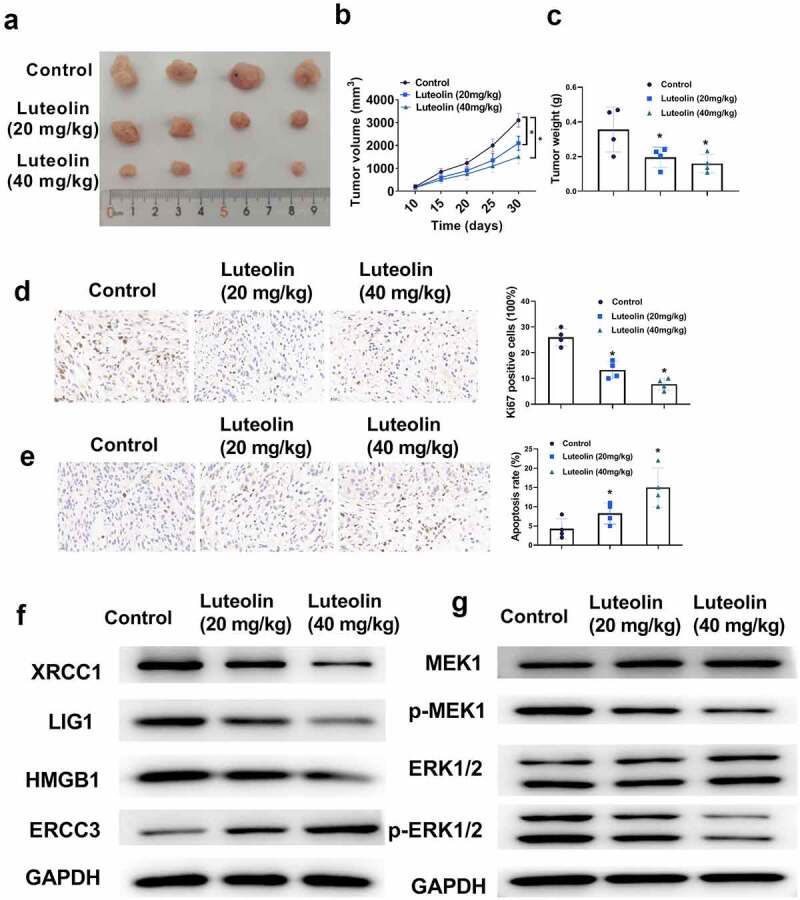
An in vivo study shows that a high dose of luteolin significantly inhibited colorectal cancer (CRC) tumourigenesis in vivo. (a) Luteolin suppresses the tumor growth of CRC in vivo. (b-c) The tumor volume and weight in the luteolin group is smaller than that in the control group. (d) Ki67-positive cells are assessed by immunohistochemical staining. (e) TUNEL analysis of the cell apoptosis of tumor sections. (f) DNA (excision) repair proteins are detected by western blotting. (g) The expressions of different proteins (p-ERK1/2, ERK1/2, MEK1, and p-MEK1) related to the MAPK signaling pathway are detected by western blotting. *P < 0.05.

A mouse model of subcutaneous CRC was successfully established. All animals were randomly assigned to three groups and treated with PBS or different doses of luteolin. Our results revealed that high luteolin doses effectively inhibited tumor growth in vivo compared with the control groups, given that significant tumor weight and volume reduction were detected in the former (Figure 6a–6c). The high-dose (40 mg/kg/d) and low-dose (20 mg/kg/d) luteolin groups presented inhibition rates of 28.31% and 40.50%, respectively, and tumor growth inhibition rates of 54.28% and 40.38%, respectively. All data were statistically different from those of the control group. Upon injection with different doses of luteolin, the number of positive cells in Ki-67 samples in luteolin group was lower than that in the control group (Figure 6d). TUNEL staining also showed that the apoptosis rate in the luteolin group was substantially higher than that in the control group (Figure 3e). Furthermore, luteolin decreased the protein levels of HMGB1, LIG, and XRCC1 but increased the level of ERCC-3 in the luteolin group in a dose-dependent manner compared with those in the control group (figure 6f). As shown in Figure 6g, western blot analysis indicated that the levels of MAPK pathway-related proteins, including MEK1 and ERK1/2, were not significantly altered, whereas the levels of p-MEK1 and p-ERK1/2 decreased in a dose-dependent manner in the luteolin group compared with those in the control group. In addition, none of the mice in the luteolin group suffered from toxic side effects, such as weight loss or activity reduction, demonstrating that luteolin could significantly change the growth profile of CRC in a xenograft model with no negative effect on daily animal activity.

## Discussion

In China, CRC ranks fifth among cancer-related deaths, and the incidence rate is increasing every year [3]. Although there are several options for the clinical treatment of CRC, the therapeutic effect is usually limited by the high recurrence rate of cancer and causes many adverse reactions, such as cardiac and neurotoxicity, renal failure, and gastrointestinal reactions [6]. In recent years, traditional Chinese medicine has aroused increasing concern in cancer treatment because of its extensive anti-tumor activity, safety, low cost, and efficiency. Gong et al. reported that curcumin could induce apoptosis and autophagy in human renal cell cancer through the Akt/mTOR pathway [35]. Wang et al. found that tetrandrine could suppress the growth of osteosarcoma by regulating the MAPK/Erk and PTEN/Akt signaling pathways [36]. In addition, numerous laboratory and epidemiological evidences suggest that the ingestion of flavonoid-rich foods reduces the risk of CRC by interfering with different stages of cancer progression, such as inhibiting carcinogenic activity, activating the carcinogenic detoxification system, and protecting DNA from oxidative stress [37]. Luteolin is a flavonoid used in traditional Chinese medicine for the treatment of hypertension, inflammatory diseases, and cancer.

The role of luteolin in CRC has been reported in several studies. Iva et al. reported that luteolin was cytotoxic to metastatic CRC SW620 cells and could activate apoptosis through FOXO3a and ERK1/2-dependent pathways [38]. Anna et al. indicated that luteolin inhibited CRC LoVo cells in a concentration-dependent manner [39]. Chan et al. reported that the viability of HCT-116 cells was remarkably decreased after luteolin treatment at 5 or 25 M, suggesting that luteolin can efficiently suppress cell growth [40]. Furthermore, a recent study reported that luteolin (7.5, 15, and 30 μM) plays a vital role in restraining the viability and tumourigenicity of HCT-116 and HT-29 cells in a concentration-dependent manner [41]. In the present study, the results indicated that luteolin decreased the viability and proliferation of HCT-116 and HT-29 cells in a dose-dependent manner, which was similar to the results of the aforementioned studies.

Luteolin can induce cell cycle arrest in the G2/M, S, and G0/G1 phases and trigger apoptosis, blocking the progression of cancer cells in vitro and in vivo, including the CRC cell line HT-29 [42]. In the present study, luteolin increased the apoptosis of HCT-116 and HT-29 cells and induced cell cycle arrest in the G2 phase, which confirmed the aforementioned findings. Cancer cells exposed to chemotherapy drugs can cause DNA damage, commonly DNA single-strand breaks, which are crucial factors in luteolin-induced apoptosis [43]. Our results in HCT-116 and HT-29 cells exposed to luteolin support the aforementioned conclusion regarding DNA damage induction. Meanwhile, the level of apoptosis was gradually elevated in luteolin-treated cells; luteolin led to an increase in the protein expression of BAX and cleaved caspase-3 and resulted in a dramatic decrease in BCL-2 expression. Cisplatin, one of the most effective chemotherapy drugs, has already been proven to be effective for treating a variety of malignant tumors, including CRC [44]. The anticancer effect of cisplatin is that it can interfere with DNA repair mechanisms, lead to DNA damage, and induce apoptosis in cancer cells [44]. In our study, cisplatin alone remarkably decreased the survival rate and increased the apoptosis rate of HCT-116 and HT-29 cells, which were further aggravated by co-treatment with luteolin in a dose-dependent manner. On account of the aforementioned results, it suggests that luteolin induced cell cycle arrest and DNA damage, thus leading to cell apoptosis by regulating the expressions of BCL-2, BAX, and caspase-3.

MAPKs belong to an extremely conservative family of serine/threonine kinases. Four different cascades share the generic MAPK signaling pathway (also called the RAF/ERK/MEK pathway), containing p38-MAPK, extracellular signal-related kinases (ERK1/2), mitogen-activated protein kinase (MEK), and Jun amino-terminal kinases (JNK1/2/3) [45]. The MAPK pathway can transform various extracellular signals into intracellular reactions via a series of phosphorylation cascades. ERK1/2 is activated upon phosphorylation by MEK1/MEK2 and is related to cell differentiation, proliferation, and cycle [46]. In the present study, the levels of MEK1 and ERK1/2 were not significantly altered, whereas those of p-MEK1 and p-ERK1/2 were decreased in a dose-dependent manner in luteolin-treated HCT-116 cells. Selumetinib is regarded as a second-generation inhibitor of MEK1 and is currently used in clinical trials for the treatment of several solid malignant tumors. We found that pre-incubation with an MEK1/2 inhibitor (selumetinib) significantly decreased the level of the DNA repair protein XRCC1 in luteolin-induced HCT-116 cells, thus leading to DNA damage. On account of the aforementioned results, it suggests that luteolin could regulate the phosphorylation of the MAPK pathway, thus being involved in the behavior of CRC cells. In the further. we will continue to carry out animal model experiments to study the pharmacodynamics of luteolin in vivo.

## Conclusion

Luteolin could suppress cell proliferation, block the cell cycle, and induce DNA damage and apoptosis progression in CRC cells by mediating the MAPK pathway. Our results indicate that luteolin, a potential anti-tumor drug, may be an effective supplement for the treatment of CRC in the future.

## Supplementary Material

Supplemental Material

Supplemental Material

## Data Availability

The datasets used and analyzed during the current study are available from the corresponding author on reasonable request.

## Abbreviations

Cell Counting Kit-8 (CCK-8), colorectal cancer (CRC), extracellular signal-related kinases (ERK1/2), Jun amino-terminal kinases (JNK1/2/3), mitogen-activated protein kinase (MEK), mitogen-activated protein kinases (MAPKs)
