# Supplementary material for: Luteolin impacts deoxyribonucleic acid repair by modulating the mitogen-activated protein kinase pathway in colorectal cancer
Source: Bioengineered. 2022 Apr 27;13(4):10998–1011. doi: 10.1080/21655979.2022.2066926 (PMC9161897; doi:10.1080/21655979.2022.2066926)
Supplement: Supplemental Material [file KBIE_A_2066926_SM7407.pdf]

editage

Editing Certificate

This document certifies that the manuscript listed below has been edited to ensure language and grammar accuracy and is error free in these aspects. The edit was performed by professional editors at Editage, a division of Cactus Communications. The author's core research ideas were not altered in any way during the editing process. The quality of the edit has been guaranteed, with the assumption that our suggested changes have been accepted and the text has not been further altered without the knowledge of our editors.

MANUSCRIPT TITLE

Luteolin impacts deoxyribonucleic acid repair by modulating the mitogen-activated protein kinase pathway in colorectal cancer

AUTHORS

Yelin Song, Jie Yu, LingLing Li, Lei Wang, Liangle Dong, Guangmin Xi, Yun Jing Lu Zuowei Li

ISSUED ON

March 14, 2022

JOB CODE

LIUOW_1

THE EDITAGE Promise QUALITY GUARANTEE

Vikas Narang

Vikas Narang
Chief Operating Officer - Editage

editage

CACTUS

Editage, a brand of Cactus Communications, offers professional English language editing and publication support services to authors engaged in over 1300 areas of research. Through its community of experienced editors, which includes doctors, engineers, published scientists, and researchers with peer review experience, Editage has successfully helped authors get published in internationally reputed journals. Authors who work with Editage are guaranteed excellent language quality and timely delivery.

impact.science

researcher.life

lifesciences.cactusglobal.com

GLOBAL :
+1(833) 979-0061 | request@editage.com

CHINA :
400-120-3020 | fabiao@editage.cn
