# Supplementary figures and images for: Luteolin impacts deoxyribonucleic acid repair by modulating the mitogen-activated protein kinase pathway in colorectal cancer
Source: Bioengineered. 2022 Apr 27;13(4):10998–1011. doi: 10.1080/21655979.2022.2066926 (PMC9161897; doi:10.1080/21655979.2022.2066926)

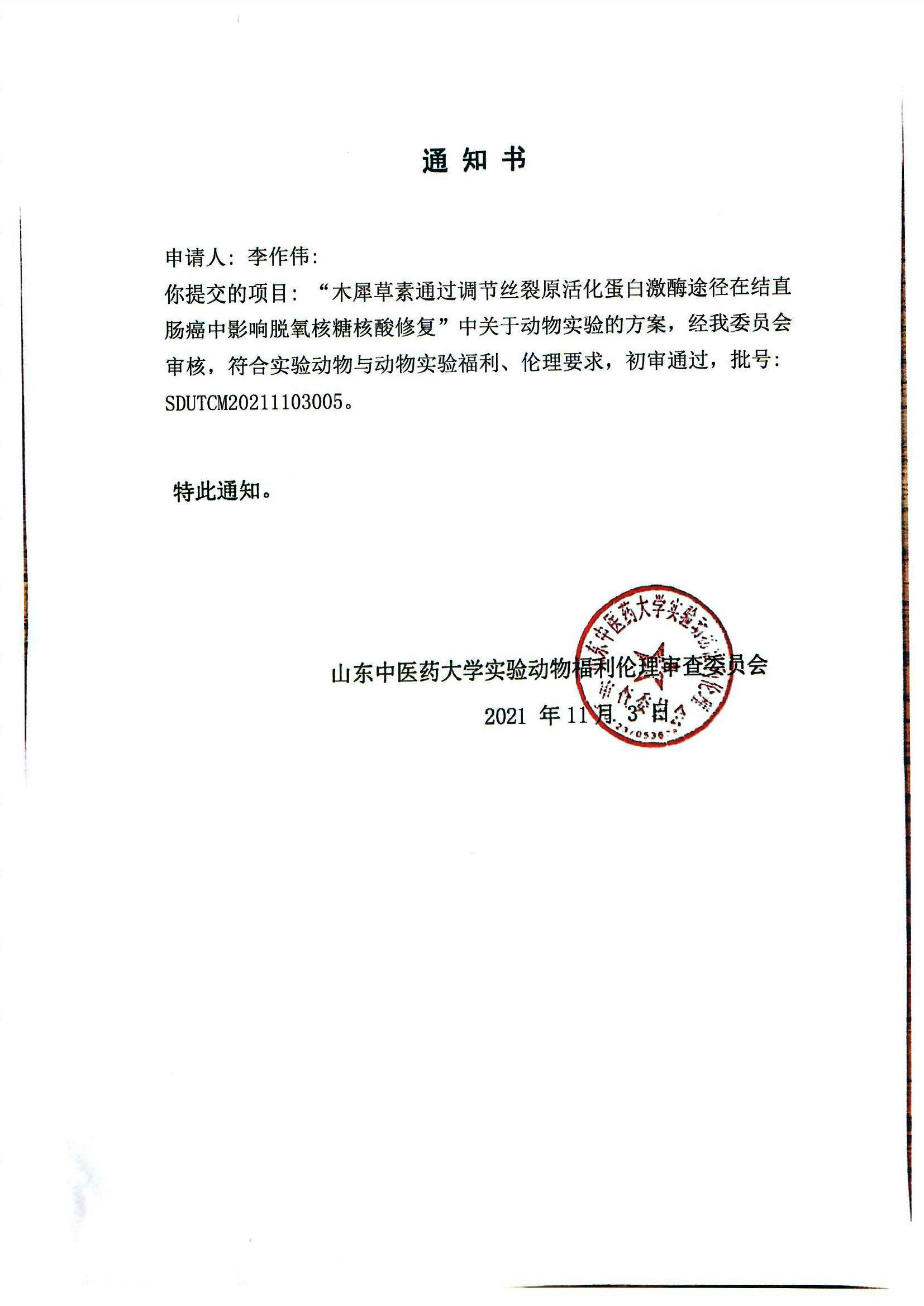

Supplement: Supplemental Material [file KBIE_A_2066926_SM0301.jpg]
